# Sex-difference in the association between social drinking, structural brain aging and cognitive function in older individuals free of cognitive impairment

**DOI:** 10.3389/fpsyt.2024.1235171

**Published:** 2024-04-08

**Authors:** Osama A. Abulseoud, Elisabeth C. Caparelli, Janina Krell‐Roesch, Yonas E. Geda, Thomas J. Ross, Yihong Yang

**Affiliations:** ^1^ Department of Psychiatry and Psychology, Mayo Clinic, Phoenix, AZ, United States; ^2^ Department of Neuroscience, Graduate School of Biomedical Sciences, Mayo Clinic College of Medicine, Phoenix, AZ, United States; ^3^ Neuroimaging Research Branch, National Institute on Drug Abuse, National Institutes of Health, Baltimore, MD, United States; ^4^ Department of Quantitative Health Sciences, Mayo Clinic Rochester, Rochester, MN, United States; ^5^ Institute of Sports and Sports Science, Karlsruhe Institute of Technology, Karlsruhe, Germany; ^6^ Department of Neurology, and the Franke Barrow Global Neuroscience Education Center, Barrow Neurological Institute, Phoenix, AZ, United States

**Keywords:** social drinking, brain age estimating, cognitive function, sex difference, alcohol

## Abstract

**Background:**

We investigated a potential sex difference in the relationship between alcohol consumption, brain age gap and cognitive function in older adults without cognitive impairment from the population-based Mayo Clinic Study of Aging.

**Methods:**

Self-reported alcohol consumption was collected using the food-frequency questionnaire. A battery of cognitive testing assessed performance in four different domains: attention, memory, language, and visuospatial. Brain magnetic resonance imaging (MRI) was conducted using 3-T scanners (Signa; GE Healthcare). Brain age was estimated using the Brain-Age Regression Analysis and Computational Utility Software (BARACUS). We calculated the brain age gap as the difference between predicted brain age and chronological age.

**Results:**

The sample consisted of 269 participants [55% men (n=148) and 45% women (n=121) with a mean age of 79.2 ± 4.6 and 79.5 ± 4.7 years respectively]. Women had significantly better performance compared to men in memory, (1.12 ± 0.87 vs 0.57 ± 0.89, P<0.0001) language (0.66 ± 0.8 vs 0.33 ± 0.72, P=0.0006) and attention (0.79 ± 0.87 vs 0.39 ± 0.83, P=0.0002) z-scores. Men scored higher in visuospatial skills (0.71 ± 0.91 vs 0.44 ± 0.90, P=0.016). Compared to participants who reported zero alcohol drinking (n=121), those who reported alcohol consumption over the year prior to study enrollment (n=148) scored significantly higher in all four cognitive domains [memory: F_3,268_ = 5.257, P=0.002, Language: F_3,258_ = 12.047, P<0.001, Attention: F_3,260_ = 22.036, P<0.001, and Visuospatial: F_3,261 _= 9.326, P<0.001] after correcting for age and years of education. In addition, we found a significant positive correlation between alcohol consumption and the brain age gap (P=0.03). *Post hoc* regression analysis for each sex with language z-score revealed a significant negative correlation between brain age gap and language z-scores in women only (P=0.008).

**Conclusion:**

Among older adults who report alcohol drinking, there is a positive association between higher average daily alcohol consumption and accelerated brain aging despite the fact that drinkers had better cognitive performance compared to zero drinkers. In women only, accelerated brain aging is associated with worse performance in language cognitive domain. Older adult women seem to be vulnerable to the negative effects of alcohol on brain structure and on certain cognitive functions.

## 1 Introduction

Recent epidemiological studies document substantial growth in the older adult population in the United States and worldwide (1, 2). Older women seem to enjoy more healthy aging compared to men (3), who tend to have a higher prevalence of mild cognitive impairment (MCI) (4). However, women tend to live longer and the prevalence of all-type dementia in women aged 60 to 69 years is almost double that in men (5). Chronic heavy alcohol drinking is a known risk factor for MCI (6) and for the transition from MCI to dementia (7).

Recent data show robust increase in the prevalence of excessive alcohol drinking among women (8–12). Women exhibit more sensitivity to alcohol; with sooner progression to alcohol-related diseases at less drinking levels compared to men (13–15). The deleterious effects of excessive alcohol drinking on cognitive functions are well documented (16–19) specifically in women (20, 21). However, the association between social drinking and the risk of cognitive decline remains controversial (22–24). Social drinking refers to drinking patterns that are accepted by the society in which they occur and has often been confused with the concept of moderate drinking which may be defined as drinking that does not generally cause problems, either for the drinker or for society (25, 26).

While several large-scale studies in elderly population suggest that moderate drinking could be associated with better cognitive performance compared to non-drinkers (6, 27–40), other studies found no cognitive benefit for alcohol drinking (41, 42) and no significant correlation between alcohol consumption and cognitive performance (43, 44). We have recently reported that low-level alcohol consumption is associated with premature brain aging (45). Others have reported similar findings in patients with alcohol use disorder (46).

Premature, advanced, or accelerated brain aging are terms used to describe the gap between chronological age and estimated brain age derived from different machine learning algorithms. Accelerated brain aging is a non-specific observation that has been reported in many conditions such as major depression (47–50), psychosis (51, 52), obsessive compulsive disorder (53), traumatic brain injury (54, 55), tobacco smoking (56), diabetes mellitus (57, 58), glioma patients following radiotherapy (59), and in healthy individuals with inadequate sleep (60). Few studies have reported negative association between accelerated brain aging and cognitive performance in patients with major psychoses (51), in older patients with depressive symptoms (61) and in patients with traumatic brain injury (54).

The relationship between casual drinking, brain aging, and cognitive function in older men and women received little attention despite rich literature documenting sex differences in brain morphology (62–66), and the effect of alcohol on brain structure (67, 68) and cognitive performance (21). Therefore, this study aimed to investigate the relationship between alcohol consumption, brain age gap and cognitive function in older adults free of cognitive impairment from the Mayo Clinic Study of Aging (MCA). We hypothesized that the degree of alcohol drinking will directly correlate with the brain age gap and the relationship between brain age gap and certain cognitive domains will be sex dependent.

## 2 Methods

### 2.1 Study setting

The study was conducted in the setting of the population-based Mayo Clinic Study of Aging (MCSA) in Olmsted County, Minnesota. The Mayo Clinic Study of Aging (MCSA) is a population-based study that utilized the medical records-linkage system to construct a sampling frame of Olmsted County residents aged 70–89 on October 1, 2004, who had been in contact with the system at least once within the 3 years prior to the index date. Duplicate records for the same persons were identified and excluded and a single record was created for each resident. Addresses of residences located on the border of Olmsted County were manually checked by a nurse abstractor to exclude subjects who were nonresidents and unique individuals were enumerated in the study sampling frame. The medical records of subjects selected for the study that were archived by the records-linkage system were screened to identify dementia. If a diagnosis of dementia was identified or suspected, the complete medical record was reviewed by a behavioral neurologist to confirm the diagnosis of dementia. Persons with a confirmed diagnosis of dementia were not contacted for participation. Subjects presumed to be free of dementia were sent a letter of invitation that briefly described the rationale and purpose of the study. This letter of invitation was followed by a recruitment telephone call, and finally an in-person evaluation or a telephone assessment. Both the study participant and informant are interviewed (69). Current or previous symptoms of anxiety, depression, or alcoholism were assessed using screening instruments such as the Patient Health Questionnaire version of the PRIME MD (70), the Beck Depression Inventory (71) and the Beck Anxiety Inventory (72). Subjects who screened positive for psychiatric symptoms were assessed further by a physician using the Clinician Evaluation Guideline component of the PRIME MD, a semistructured interview that generates Diagnostic and Statistical Manual of Mental Disorders – IV (DSM-IV) (73) diagnoses for depressive disorders, anxiety disorders or alcohol use disorders (70). The details of study design, sampling and measures have been described in the Methodology paper of the Mayo Clinic Study of aging (69). For this analysis, we considered participants aged 70 years and older and free of cognitive impairment. Enrolled participants underwent a comprehensive in-person evaluation including the Clinical Dementia Rating Scale, alcohol consumption pattern over the past year, a neurological evaluation and neuropsychological testing to assess four cognitive domains: memory, executive function, language, and visuospatial skills (4, 69). Data included in this analysis was collected between March 2006 and March 2022.

### 2.2 Alcohol consumption history

Participants completed a 128-item food-frequency questionnaire (74) which is a modification of the Block 1995 Revision of the Health Habits and History Questionnaire (75). The questionnaire included 25 beverages and focused on usual drinking habits during the year prior to enrollment. Alcoholic beverages were categorized into four groups: red wine, white wine, beer, and liquor. Participants were asked to provide the average number of servings per month in each category over the year prior to study enrollment. Ethanol content in each serving was calculated as follows: For red wine [volume per serving = 147.9 mL* ethanol concentration per mL = 0.106 gm], for white wine [volume per serving = 147.9 mL* ethanol concentration per mL = 0.103 gm], for beer [volume per serving = 354.9 mL* ethanol concentration per mL = 0.039 gm], and for liquor [volume per serving = 44.4 mL* ethanol concentration per mL = 0.347 gm]. Total monthly ethanol consumption was calculated by summing all consumed alcohol in all four categories. Finally, average daily ethanol intake (in gm) was obtained by dividing the monthly consumption over 30 days.

### 2.3 Cognitive testing

All the participants included in the study are cognitively normal older adults, and their Clinical Dementia Rating (CDR) score is 0. The Methodology paper of Mayo Clinic Study of Aging (69) describes, in detail, the protocol we follow in classifying a person to be cognitively normal or having Mild Cognitive Impairment or dementia based on published criteria. A weekly multidisciplinary consensus panel classifies a person to be cognitively unimpaired, or MCI or dementia based on published criteria.

Neuropsychological testing was performed using nine validated tests to assess impairment in four domains (A) attention using (1) Trail Making Test B (76) and (2) Digit Symbol Substitution Test (77); (B) language using (3) Boston Naming Test (78) and (4) Category Fluency (79); (C) memory using (5) Logical Memory-II (delayed recall), (6) Visual Reproduction-II (delayed recall) (80) and (7) Auditory Verbal Learning Test (delayed recall) (81), and (D) visuospatial using (8) Picture Completion and (9) Block Design (77). The raw scores on each test were transformed into an age-adjusted score using normative data from Mayo’s Older American Normative Studies and were scaled to have a mean of 10 and a SD of 3 (81). Domain scores were computed by summing the adjusted and scaled scores within a domain and scaling the combined scores to allow comparisons across domains (4, 69, 82).

### 2.4 Severity weighted sum of disease

Medical comorbidity was measured using the Charlson index, which is a widely used weighted index that takes into account the number and severity of diseases (83).

### 2.5 MRI data acquisition

Magnetic resonance imaging (MRI) was performed using 3-T scanners (Signa; GE Healthcare) equipped with an 8-channel phased array coil (GE Healthcare). A 3-dimensional magnetization–prepared rapid gradient echo sequence was performed (84, 85) with the following sequence: TR = 200 ms, TE = 20 ms, section thickness = 3.3 mm with 49 sections, flip angle = 20° and in-plane matrix of 256 × 224 (86). All images have the following resolution (x,y,z)= 1.016 × 1.016 × 1.200 mm^3^. Images were corrected for distortion due to gradient nonlinearity and for bias field (87).

### 2.6 Brain age prediction

The Brain-Age Regression Analysis and Computational Utility Software (BARACUS version 1.1.4; https://github.com/BIDS-Apps/baracus (88)) was used to generate each participant’s brain age prediction. The software requires a T1-weighted structural MRI to compute the brain age prediction. In the first step, the software processes the structural MRI using FreeSurfer version 5.3. The FreeSurfer uses three atlas: Desikan-Killiany Atlas, DKT Atlas and Destrieux Atlas (CorticalParcellation - Free Surfer Wiki (harvard.edu)) to generate its output. The FreeSurfer output is then used to generate the brain age prediction, with several model options. The prediction model was trained on the BARACUS database (default model: Liem2016:OCI_norm). For more information on the prediction model, see Liem et al. (89). Scans were processed using the computational resources of the NIH High Performance Computing (HPC) Biowulf cluster (http://hpc.nih.gov).

### 2.7 Statistical analyses

#### 2.7.1 Characterization measures

Clinical characterization, alcohol consumption and performance in cognitive domains (z-scores) were first tested for normality using Shapiro-Wilk’s test. Data are shown as mean ( ± SD) if normally distributed and as median and interquartile range if not normally distributed. Unpaired t-tests or Mann Whitney U tests were used to compare clinical variables’ means or medians respectively between men and women and between drinkers and zero-drinkers. We used univariate analysis with age and years of education as covariates to compare performance in cognitive tests between drinkers and zero-drinkers.

#### 2.7.2 Age gap

The brain age gap was calculated by subtracting the chronological age from *the* predicted age derived from BARACUS. Further analyses were conducted in R (version 4.2.0; (90)). To ensure generalizability of BARACUS to our sample, we conducted a Pearson correlation analysis comparing the predicted brain age to the chronological age. The relationship between the brain age gap, average total daily ethanol (gm) consumed over the preceding year, and cognitive function domain z-scores was assessed using linear regression via the *lm* function in the stats package for R (90). We included chronological age, chronological age squared (because the relationship between predicted and chronological age is not perfectly linear (89), sex, years of education, smoke status, BMI, and comorbid medical condition (severity weighted sum of disease) as covariates for the regression models. Participants who reported zero alcohol consumption over the past 12 months were excluded from this analysis. *Post hoc* regression analysis for each sex was then used to untangle a potential sex difference on the effect of alcohol consumption on brain age gap and cognitive function.

Results from the statistical analyses were considered statistically significant at P<0.05.

## 3 Results

### 3.1 Demographics

A total of 269 participants were sorted into two groups based on their sex with 55% (n=148) men and 45% women (n=121). Both groups did not display statistically significant differences in terms of their median age at time of MRI [median (IQ range): 78.6 (7.6) in men vs 78.2 (7.0) in women], age at time of cognitive testing, body mass index (BMI), or severity weighted sum of diseases. Significantly more men reported history of smoking (47.3% in men vs 35.5% in women). Fifty five percent of participants reported alcohol drinking and 45% reported zero alcohol consumption over the past over the past 12 months. Compared to zero drinkers, drinkers were younger (P=0.002), had significantly more years of education (P=0.009). Significantly more drinkers reported smoking (47.9% in drinkers vs 42% in zero drinkers), but scored lower in the severity weighted sum of diseases (P=0.02). See **Table 1** for more details.

**Table 1:** Demographics and drinking status over the past 12 months.

		All (n=269)	Men (n=148)	Women(n=121)	Sex difference (P value)	Zero drinkers (n=121)	Drinkers (n=148)	Drinking status difference (P value)
Age at MRI (years)	Median	78.6	78.150	79.100	0.66	79.700	77.550	0.002
	Min- Max	72.1-90.1	72.1-90.1	72.1-90.1		72.3-90.1	72.1-90.1	
	Interquartile Range	7.6	7.0	7.7		7.4	7.4	
Age at FFQ (years)	Median	77.5	77.500	77.800	0.66	78.900	76.450	0.006
	Min- Max	71.8-90.1	72-90.1	71.8-90.1		72-90.1	71.8-90.1	
	Interquartile Range	7.6	7.3	8.0		7.9	6.8	
Time between FFQ and MRI (years	Median	0.6	0.53	0.69	0.54	0.66	0.60	0.400
	Min- Max	0.0-8.4	0.0-5.1	0.0-8.4		0.04-8.4	0.0-5.1	
	Interquartile Range	1.0	1.07	1.05		1.15	0.97	
Years of education	Median	14.0	14.00	14.00	0.16	13.00	14.50	0.009
	Min- Max	11-20	11-20	11-20		11-20	11-20	
	Interquartile Range	4.0	6	4		4	5	
BMI (Kg/m2)	Median	27.2	27.3250	26.9900	0.72	27.0100	27.2150	0.600
	Min- Max	18.0-43.8	19.1-43.8	18.0-42.6		19.6-42.6	18.0-43.8	
	Interquartile Range	5.8	5.65	6.98		5.57	6.19	
Smoking history [N(%)]		113 (42%)	70 (47.3%)	43 (35.5%)	<0.0001	42 (34.7%)	71 (47.9%)	0.03
Severity weighted sum of diseases	Median	3.0	3.00	2.00	0.20	4.00	2.00	0.024
	Min- Max	0.0-16	0.0-16	0.0-14		0.0-14	0.0-16	
	Interquartile Range	4.0	4	3		4	3	

MRI, magnetic resonance imaging; FFQ, food frequency questionnaire; BMI, body mass index.

### 3.2 Performance in cognitive tests

All four cognitive domains had highly significant intercorrelations (P<0.001 each) as shown in **Supplementary Table S1**. Compared to men, women participants scored significantly higher in all cognitive domains [memory z-score (mean ± SD: 1.12 ± 0.87 in women vs 0.57 ± 0.89 in men), language z-score (mean ± SD: 0.66 ± 0.8 in women vs 0.33 ± 0.72 in men), and attention z-score (mean ± SD: 0.79 ± 0.87 in women vs 0.39 ± 0.83 in men)] except in visuospatial tasks where men performed better than women (mean ± SD: 0.71 ± 0.91 in men vs 0.44 ± 0.90 in women). Comparing zero drinkers with drinkers shows that drinkers scored significantly higher in language (mean ± SD: 0.57 ± 0.74 in drinkers vs 0.37 ± 0.79 in zero drinkers), and attention (mean ± SD: 0.7 ± 0.75 in drinkers vs 0.42 ± 0.98in zero drinkers). No significant differences were observed between zero drinkers and drinkers in memory or visuospatial domains. Since the alcohol drinking group were significantly younger with more years of education compared to the non-alcohol drinkers, we repeated the analysis using both age and years of education as covariates. The results show drinkers performed better in all four cognitive domains [memory: F_3,268 _= 5.257, P=0.002, Language: F_3,258 _= 12.047, P<0.001, Attention: F_3,260_ = 22.036, P<0.001, and Visuospatial: F_3,261_ = 9.326, P<0.001] (**Table 2**).

**Table 2:** Cognitive performance results.

		All (n=269)	Males (n=148)	Females (n=121)	Sex difference (P value)	Zero drinkers (n=121)	Drinkers (n=148)	Drinking status difference (P value)	Drinking status difference corrected for age and years of education
Performance in cognitive function domains (z-scored)	Memory (mean ± SD)	0.82 ± 0.92	0.57 ± 0.89	1.12 ± 0.87	<0.0001	0.8 ± 0.93	0.83 ± 0.92	P=0.8	F_3,268 = _5.257, P=0.002
	Language (mean ± SD)	0.48 ± 0.77	0.33 ± 0.72	0.66 ± 0.8	0.0006	0.37 ± 0.79	0.57 ± 0.74	P=0.03	F_3,258 = _12.047, P<0.001
	Attention (mean ± SD)	0.57 ± 0.87	0.39 ± 0.83	0.79 ± 0.87	0.0002	0.42 ± 0.98	0.7 ± 0.75	P=0.007	F_3,260 = _22.036, P<0.001
	Visuospatial (mean ± SD)	0.59 ± 0.91	0.71 ± 0.91	0.44 ± 0.90	0.016	0.48 ± 0.85	0.69 ± 0.96	P=0.06	F_3,261 = _9.326, P<0.001

### 3.3 Sex differences in demographics, alcohol consumption and performance in cognitive tests among individuals who reported alcohol drinking over the year prior to enrollment

Among participants who reported alcohol drinking over the past year, 58% (n=86) were men and 42% (n=62) were women. No differences between the two groups were observed in median age at time of MRI, years of education, BMI, smoking history, or severity weighted sum of diseases. men alcohol drinkers reported consuming more average daily beer [median (IQ range): 0.83 (3.7) g/d in men vs 0.0 (0.83) g/d in women) and more total average daily alcohol [median (IQ range): 7.58 (14.75) g/d in men vs 4.15 (7.58) g/d in women]. Women reported drinking more white wine compared to men [median (IQ range): 0.46 (1.33) g/d in women vs 0.0 (0.91) g/d in men]. Compared to men, women participants who reported alcohol drinking over the past year had better memory (mean ± SD: 1.12 ± 0.86 in women vs 0.61 ± 0.9 in men), language (mean ± SD: 0.74 ± 0.76 in women vs 0.45 ± 0.7 in men), and attention (mean ± SD: 0.92 ± 0.78 in women vs 0.54 ± 0.69 in men). Men performed better in visuospatial domain (mean ± SD: 0.87 ± 0.89 in men vs 0.44 ± 1.01 in women) (**Table 3**).

**Table 3:** Demographics, average daily drinking and cognitive performance in participants who reported alcohol drinking over the past 12 months.

			Alcohol drinkers		Sex difference (P value)
			Men (n=86)	Women (n=62)	
Age at MRI (years)		Median	77.700	77.000	0.9
		Min- Max	72.1-90.1	72.1-90.1	
		Interquartile Range	6.7	8.2	
Age at FFQ (years)		Median	76.850	76.200	0.6
		Min- Max	72.0-90.1	71.8-89.3	
		Interquartile Range	6.4	8.1	
Time between FFQ and MRI (years)		Median	0.57	0.63	0.6
		Min- Max	0.0-5.1	0.0-3.8	
		Interquartile Range	1.05	0.70	
Years of education		Median	15.00	14.50	0.5
		Min- Max	11.0-20.0	11.0-20.0	
		Interquartile Range	6	3	
BMI (Kg/m2)		Median	27.5400	26.9600	0.1
		Min- Max	19.06-43.8	18.0-42.4	
		Interquartile Range	6.04	6.18	
Severity weighted sum of diseases		Median	2.50	2.00	0.4
		Min- Max	0.0-16.0	0.0-13.0	
		Interquartile Range	4	3	
Average alcohol consumption over the past 12 months (g/d)	Red wine	Median	0.00	0.24	0.6
		Min- Max	0.0-18.3	0.0-15.7	
		Interquartile Range	1.37	1.10	
	White wine	Median	0.00	0.46	0.001
		Min- Max	0.0-11.9	0.0-11.9	
		Interquartile Range	0.91	1.33	
	Beer	Median	0.83	0.00	<0.0001
		Min- Max	0.0-62.3	0.0-34.6	
		Interquartile Range	3.70	0.83	
	Liquor	Median	0.23	0.00	0.1
		Min- Max	0.0-38.5	0.0-38.5	
		Interquartile Range	4.14	1.35	
	Total	Median	7.58	4.15	0.03
		Min- Max	0.5-62.3	0.5-39.4	
		Interquartile Range	14.75	7.58	
Performance in cognitive function domains (z-scored)	Memory (mean ± SD)		0.61 ± 0.9	1.12 ± 0.86	0.0007
	Language (mean ± SD)		0.45 ± 0.7***	0.74 ± 0.76*	0.018
	Attention (mean ± SD)		0.54 ± 0.69**	0.92 ± 0.78	0.001
	Visuospatial (mean ± SD)		0.87 ± 0.89**	0.44 ± 1.01	0.007

Missing 1 participant (*), 3 subjects (**), 5 subjects (***).

### 3.4 Testing the utility of BARACUS in predicting brain age in this cohort of elderly individuals

Since the mean age of our study participants is 79.4 ± 4.7 years, which is significantly older compared to the training cohort used for BARACUS development, we examined the correlation between chronological age and brain age in all four outputs. Stacked anatomy had an intrinsic ceiling effect at age 75.6 years (**Supplementary Figure S1**) as previously reported (89). Fortunately, the other three prediction outputs (cortical area, cortical thickness, or subcortical volume) predicted brain age with robust correlations (**Supplementary Figures S2A–C**), so those were included in further analysis. We calculated brain age gap as the difference between predicted brain age and chronological age.

### 3.5 The association between brain age gaps and average alcohol consumption over the past 12 month

For this analysis, we excluded from the original cohort (n=269) participants that reported zero alcohol consumption over the past year (n=121), leading to a final sample of 148. Besides the covariates mentioned in the methods section, interaction of sex and alcohol consumption was also included in each model. Significant positive association was observed only between the age gap, calculated from the predicted age “cortical thickness”, and alcohol consumption (*β* = 0.2, P = 0.03). However, the association of age gap and alcohol consumption was not significant when age gap was calculated either from “cortical area” (*β* = 0.02, P = 0.79) or from “subcortical volume” (*β* = 0.13, P = 0.30). For this reason, the age gap calculated from “cortical thickness” was the only one considered for further analysis.

### 3.6 The association between average alcohol consumption over the past year, brain age gap and cognitive function z-scores

Using cortical thickness prediction output, we examined the correlation between brain age gap and alcohol consumption with cognitive measures for the cohort of 148 participants who reported alcohol consumption on the past 12 month. For this purpose, the z-scores of four different cognitive domains: memory, language, attention and visuospatial were used in four different models. In addition to the covariates mentioned in the methods, interaction of sex and alcohol consumption and of sex and the respective cognitive measures were also included in each model. We found significant positive correlation between alcohol consumption and brain age gap in all four models: memory (*β* = 0.20, P=0.033), attention (*β* = 0.20, P=0.038), Visual spatial (*β* = 0.20, P=0.039) and language (*β* = 0.19, P=0.037). However, only language domain z-score showed significant correlation with age gap (*β* = -1.92, P=0.0454). In addition, we also found significant association between age gap and language-sex interaction (*β* = 2.91, P=0.0261).

**Figure 1:**
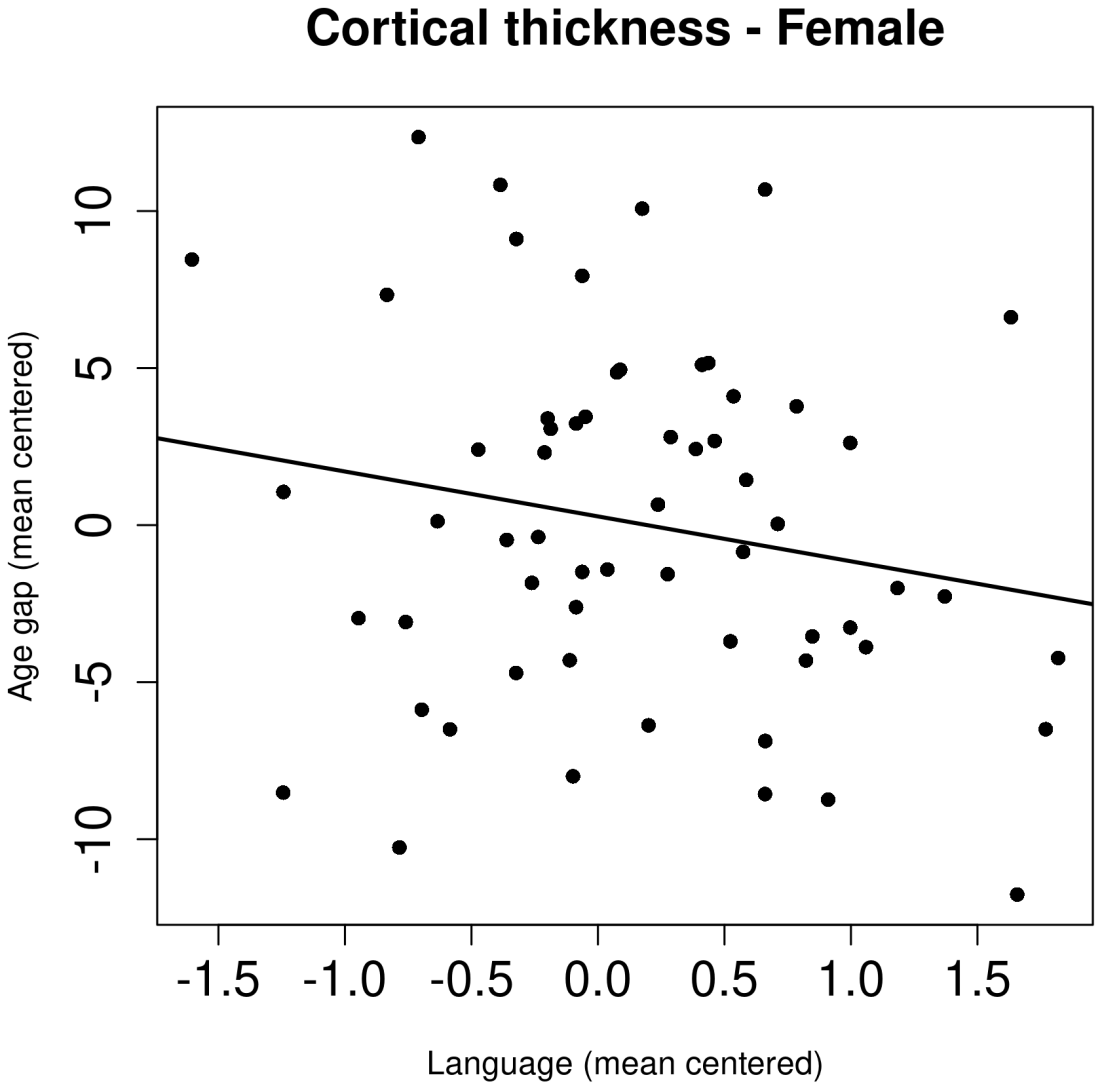
The association between brain age gap, calculated from cortical thickness, and language z-score in female participants. The age gap and language z-score were mean-centered.

*Post hoc* regression analysis for each sex (82 men and 61 women), of age gap with alcohol consumption and language z-score, including the covariates mentioned in the methods, revealed significant negative correlation between brain age gap and language z-scores in women only (*β* = -2.54, P=0.00876, P_corr_ = 0.017 (P_corr_ = P-value corrected for multiple comparison)) (**Figure 1**).

## 4 Discussion

The results of this study lend more evidence to prior reports that women perform better in memory, language, and attention cognitive tasks (91–94), while men tend to have higher scores in visual-spatial domain (94, 95). In addition, older individuals who reported social drinking over the 12 months prior to study enrollment performed better in language and attention compared to those who reported zero drinking during the same time interval (6, 27–40). Furthermore, these data also replicate our recent results showing a direct correlation between higher daily alcohol consumption and brain age gap, i.e., accelerated brain aging (45). In addition, we show here that the relationship between brain age gap and certain cognitive functions is sex-dependent where we observed significant negative correlation between brain age gap and language z-score only in women. These results of sex-specific effect of brain age gap on cognitive function support our hypothesis and lend more evidence to the concept of women’s vulnerability to alcohol drinking in in a range of cognitive process (96). More importantly, our study and others (62–66, 97) bring more attention to the need for sex-difference studies in brain morphology differences with aging.

### 4.1 Social drinkers, compared to zero-drinkers, score higher in cognitive tasks

Our results are consistent with a large body of literature documenting that moderate alcohol consumption is associated with better cognition. Large-scale studies such as the Nurses’ Health Study (n=12,480, age range 70-81 years, with follow up at 2 years), reported that moderate (<15 g alcohol/day) drinkers had better mean cognitive scores than nondrinkers and the relative risk of a substantial decline in performance over a two-year period was significantly less [0.85 (95% CI: 0.74-0.98)] among moderate drinkers, as compared with nondrinkers (27). Similar results were found in a large-scale community-based study in Ireland, the Netherlands, and Scotland (n=5804, with 3000 women, aged 70-82 years, followed up for 3.2 years). In that study, cognitive performance was better for women drinkers than nondrinkers for all cognitive domains over the 3.2-year follow-up; no significant effects were seen for men. The rate of cognitive decline was similar for drinkers and nondrinkers for all cognitive domains, except for MMSE, which declined significantly less in women drinkers than nondrinkers (28). In contrast, the Health and Retirement Study (n= 19,887 with 11,943 women who underwent serial cognitive assessments over 12 years between 1996 and 2008) showed that low to moderate drinkers (<8 drinks/week in women and < 15 drinks/week in men), compared with never drinkers, were less likely to have a consistently low cognitive trajectory and also had decreased annual rates of total cognitive function decline (40).In addition, four heart studies [Honolulu Heart Program (n=3556 aged 71 to 93 years) (29), Caerphilly Prospective Study of Heart Disease and stroke (n=1870 men aged 55-69 years) (30), Framingham Heart Study (n=1788, aged 55-88 years) (31) Cardiovascular Health Study (n=373 cases with incident dementia and 373 controls aged 65 years and older) (32)] and the Vascular Aging Study (n=1389 aged 59-71 years) (33), all showed better cognitive performance or decreased probability of cognitive impairment or decreased odds for the incidence of dementia in moderate drinkers compared to both non-drinkers and heavy drinkers. Similar results were reported in several population-based studies (6, 34–39).

There are several, non-biological factors that could explain this intriguing observation. For example, non-drinkers could be former drinkers who stopped because of alcohol-related complications. Similarly, socioeconomic status could mediate some of the supposedly beneficial effects of alcohol on cognition since individuals with higher socioeconomic status are more likely to afford alcohol drinking compared to those with lower socioeconomic status. Years of education could be another factor.

Individuals who reported alcohol drinking (compared to zero drinkers) had more years of education and performed significantly better in all four cognitive domains. This observation could be related to the cognitive reserve hypothesis, which postulates that the brains of individuals with more years of education, leisure or mentally stimulating activities, and more complex occupations, among other factors, are more tolerant to age-related changes with less likelihood of developing mild cognitive impairment and dementia (98–100). Kaur et al. reported a significant difference in the cognitive reserve between those with normal and abnormal cognitive function in a cohort of community dwelling older adults (≥60 years, n=370) (101). In a meta-analysis by Meng and D’Arcy, individuals with lower education had a higher risk for dementia, the pooled OR was 2.61 (95%CI 2.21– 3.07) for prevalence studies and 1.88 (95%CI 1.51–2.34) for incidence studies (102). Similarly, high education was associated with a decreased rate of post-stroke dementia in a recent systematic review and meta-analysis (103).

It is important here to note that several studies did not find any cognitive benefit for alcohol drinking. In the Women’s Health Initiative Memory Study (n=4461 elderly women aged 65-79 years, followed for an average of 4.2 years), compared with no alcohol intake, intake of one or more drinks per day was associated with higher baseline Modified Mini-Mental State Examination scores (p < 0.001) and a covariate-adjusted odds ratio of 0.40 (95% confidence interval: 0.28, 0.99) was indicative of significant declines in cognitive function (41). A census-based study from Spain (n=3888, mane age = 55 years with longitudinal follow up at 2.5 and 4.5 years) reported no benefit for low-to-moderate alcohol consumption on cognitive decline. Compared to zero drinkers, the adjusted odds ratio for men with less than 12 g alcohol/day and men with 12-24 g alcohol/day and former drinkers were 0.61 (95% confidence interval (CI): 0.31, 1.20), 1.19 (95% CI: 0.61, 2.32), and 1.03 (95% CI: 0.59, 1.82), respectively. Similar results were observed in women, i.e., odds ratios for mild, moderate and former drinkers were 0.88 (95% CI: 0.45, 1.72), 2.38 (95% CI: 0.98, 5.77), and 1.03 (95% CI: 0.48, 2.23) respectively (42). One study from California examined the association of cigarette smoking and alcohol consumption at baseline with risk of poor cognitive function 13-18 years later (n=511). Among women, increasing consumption of alcohol predicted a significant decline in the long-term recall and savings scores of the visual reproduction test. Moderate drinking, approximately two drinks per day, predicted categorically defined poor performance on the Buschke long-term recall task in women. In contrast, alcohol consumption was not associated with cognitive function in men (104). Two small studies from Australia [ (43) (n=209 World War II veteran men) and (44) (n=327 aged ≥75 years)] reported no significant correlation between alcohol consumption and cognitive performance.

Given the inconsistent results and the fact that any potentially beneficial cognitive effects for moderate drinking are outweighed by increased risk for other health conditions, particularly cancer (105), clinicians should err on safety and avoid encouraging social drinking.

### 4.2 Direct correlation between average daily alcohol consumption and brain age gap

Our results of significant association between the average daily alcohol consumption and brain age gap is in agreement with our recent report of dose-dependent association between social drinking and brain age gap (45) as well as with Guggenmos et al. (46), in patients with alcohol use disorder, and with the Bostrand et al. study utilizing data from the UK Biobank where alcohol use showed a positive association with higher predicted brain age (106). Whether the alcohol-associated aging process extends beyond the brain, remains to be seen. However, recent studies report that alcohol-associated aging could be related to triggering neuroimmune responses, including pro-inflammatory cytokine release (107) which could affect telomere length. This was shown recently by Jung et al. who reported that DNA methylation derived telomere length was 0.06 kilobases shorter per year in patients with alcohol use disorder compared to healthy controls after correcting for BMI and smoking status (p = 0.002) (108). The current study brings new insight by linking brain aging to cognitive performance which is critical for functionality and overall quality of life.

### 4.3 Inverse correlation between brain age gap and language z-score in women

Our current understanding for the neuroanatomical substrate of sex difference in cognition is limited despite the rich literature documenting sex difference in brain structure (109–111). Furthermore, BARACUS and other brain age estimating algorithms do not provide data on the specific structural correlates of brain age prediction (112). Despite these limitations, one can envision a three-step approach starting by mapping the neuroanatomical locations of cognitive domains, second examining whether structural differences between men and women are reported in these locations and finally studying sex-specific vulnerability of these brain regions to alcohol. We found that brain age gap showed a significant inverse association with language performance in women. In this study, language was tested using Boston Naming (78) and Category Fluency Tests (79). Using functional MRI, Baldo et al. reported that performance on the Boston Naming Test was associated with a network consisting mainly of the left mid to posterior middle temporal gyrus and underlying white matter (113). Language lateralization shows sex difference (114) with more temporal lobe asymmetry in men. Men have larger grey matter volumes in right middle temporal gyrus (115) and alcohol dependence is associated with significant reductions in temporal lobe tissue content (116). In addition, studies have reported sex differences in the effect of aging on brain morphology. For example, men, compared to women, show greater variability in volumetric measures (62, 63), but not in cortical thickness (63). Moreover, while both men and women show decline in hippocampal volume with age (64), men tend to have steeper and significant negative correlation between age and hippocampal volume compared to women in some (65, 66), but not all studies (97). Understanding such sex differences in brain structure, may provide useful information to probe the mechanisms that underlie sex-specific vulnerabilities in mental disorders including the effect of social drinking on cognition in older adults [reviewed in (117)].

## 5 Limitations and conclusion

The results of this study should be interpreted in the light of its strengths and limitations. Our study has large, well characterized population-based sample with rigorous neuropsychological and neurological testing. However, 98% of the participants are of Caucasian descent and they have relatively highly educated. Our cross-sectional study, despite an agreement with numerous cross-sectional brains aging studies (47–60) cannot make a definitive conclusion about accelerated aging and longitudinal studies are rather needed. In addition, more men, compared to women [58.1% (86/148) vs 51.2% 52/121)] reported alcohol drinking. While this difference is not statistically significant, it could have impacted the power for identifying sex differences in the association between drinking, cognitive function, and brain age gap. All participants were ambulatory, however, the effect of physical activity on cognitive function was not the focus of this study as we, and others, have shown that physical activity may help to improve cognitive function and, consequently, delay the progression of cognitive impairment in the elderly (118–120). Carvalho et al. reviewed 27 studies and reported a significant positive correlation between physical activity and cognitive function in late life (121). In addition, MCSA did not discriminate between social drinking and moderate drinking and did not collect data on alcohol use disorder history or other addictions besides tobacco use. The time interval between brain imaging and neuropsychological testing was long (≥5 years) in few patients. Despite these limitations, our finding of a significant association between higher average daily alcohol consumption and accelerated brain aging, as well as an inverse correlation between larger brain age gap and performance in older women highlights the specific negative effect of social drinking on women.

## Data availability statement

The original contributions presented in the study are included in the article/**Supplementary Material**. Further inquiries can be directed to the corresponding author.

## Ethics statement

The studies involving humans were approved by Mayo Clinic Rochester, IRB # 14-004401. The studies were conducted in accordance with the local legislation and institutional requirements. The participants provided their written informed consent to participate in this study.

## Author contributions

OA has full access to all data in the study and take responsibility for the integrity of the data and the accuracy of the data analysis. Concept and design: OA. Acquisition, and analysis of data: OA and EC. Interpretation of data: All authors. Drafting of the manuscript: OA. Critical revision of the manuscript for important intellectual content: All authors. All authors contributed to the article and approved the submitted version.

## Supplementary material

Supplementary Figure 1 Correlation between chronological age and predicted brain age using stacked anatomy.

Supplementary Figure 2 Correlation between chronological age and predicted brain age using **(A)** cortical area, **(B)** cortical thickness, and **(C)** subcortical volume.

Supplementary Table 1 Intercorrelations (Pearson correlation r values) between different cognitive domains.
