# Supplementary figures and images for: Sex-difference in the association between social drinking, structural brain aging and cognitive function in older individuals free of cognitive impairment
Source: Front Psychiatry. 2024 Apr 8;15:1235171. doi: 10.3389/fpsyt.2024.1235171 (PMC11033502; doi:10.3389/fpsyt.2024.1235171)

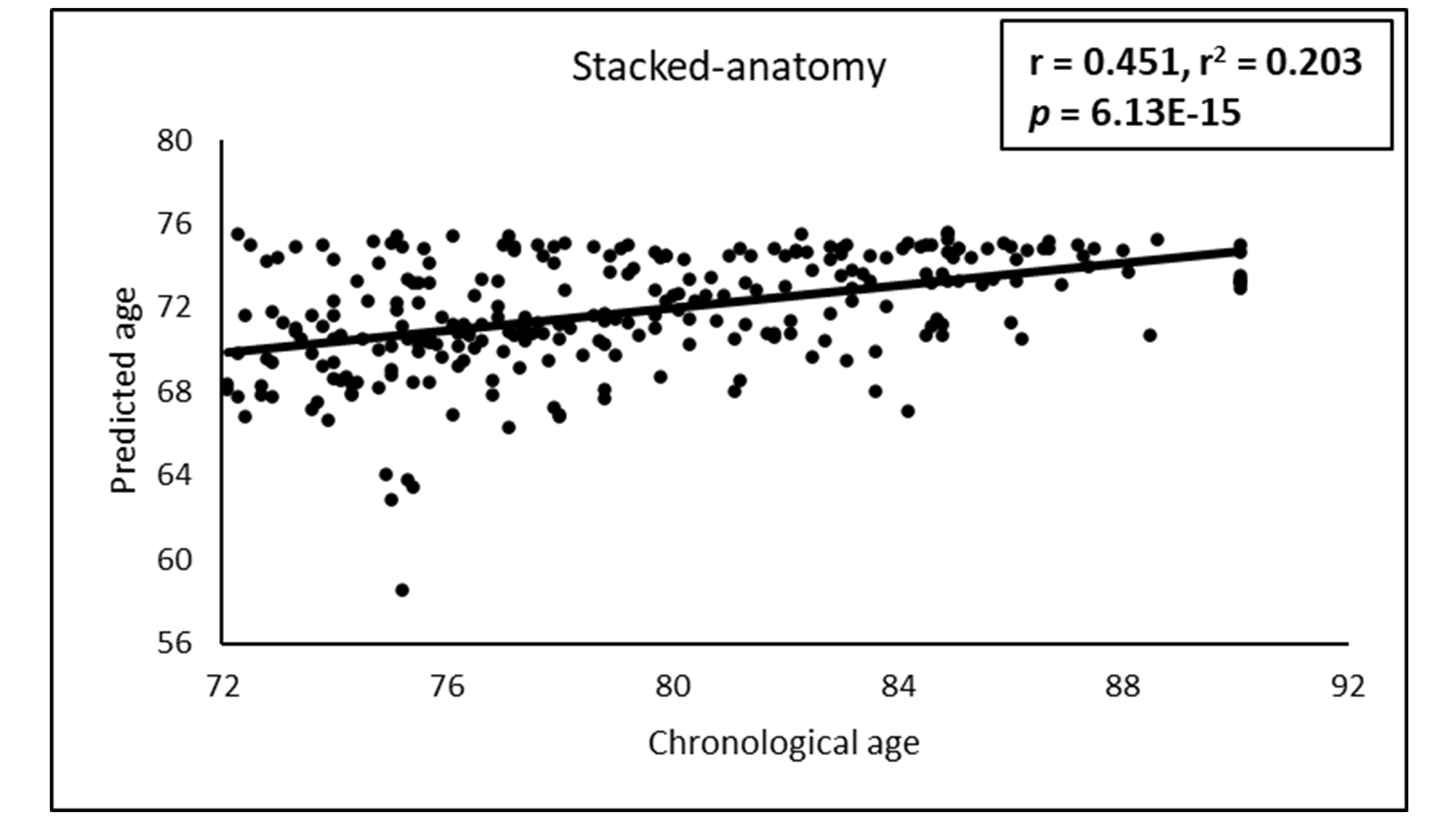

Supplement: Supplementary Figure 1 — Correlation between chronological age and predicted brain age using stacked anatomy. [file Image_1.tif]

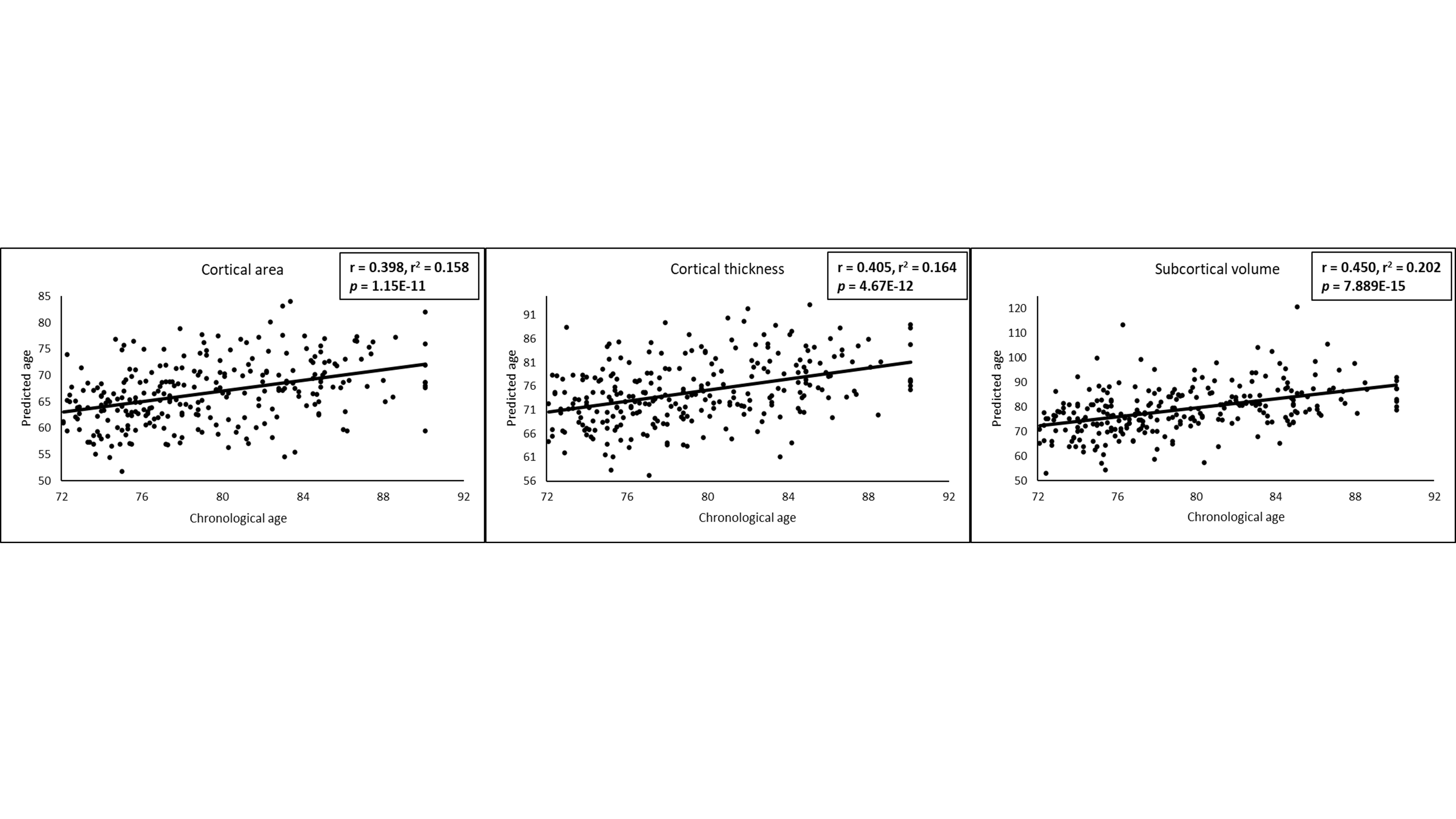

Supplement: Supplementary Figure 2 — Correlation between chronological age and predicted brain age using (A) cortical area, (B) cortical thickness, and (C) subcortical volume. [file Image_2.tif]
