# Supplementary material for: Sex-difference in the association between social drinking, structural brain aging and cognitive function in older individuals free of cognitive impairment
Source: Front Psychiatry. 2024 Apr 8;15:1235171. doi: 10.3389/fpsyt.2024.1235171 (PMC11033502; doi:10.3389/fpsyt.2024.1235171)
Supplement: Supplementary Table 1 — Intercorrelations (Pearson correlation r values) between different cognitive domains. [file Table_1.docx]

**Supplementary table S1: Correlation matrix (Peasron r values) showing highly significant intercorrelations between all four cognitive domains (P<0.001 each)**

|  | Language | Attention | Visualspatial | Memory |
| --- | --- | --- | --- | --- |
| Language | 1.00 | 0.51 | 0.34 | 0.39 |
| Attention | 0.51 | 1.00 | 0.44 | 0.43 |
| Visualspatial | 0.34 | 0.44 | 1.00 | 0.36 |
| Memory | 0.39 | 0.43 | 0.36 | 1.00 |
